# Delayed mobilization in distal humerus fractures managed with plating

**DOI:** 10.6026/973206300222371

**Published:** 2026-04-30

**Authors:** Animesh Gupta, Vineet Kumar, Raj Kumar Arya, Prabhat Kumar, Vanshika Singh

**Affiliations:** 1 Department of Orthopaedics, Dr. Ram Manohar Lohia Institute of Medical Sciences (DR.RMLIMS), Lucknow, Uttar Pradesh, India

**Keywords:** Distal humerus fractures, delayed mobilization, pre-contoured locking plates, Mayo Elbow Performance Score (MEPS), functional outcomes

## Abstract

The optimal timing of postoperative mobilization following surgical fixation of distal humerus fractures remains controversial, particularly in complex intra-articular fractures where concerns regarding fixation stability and elbow stiffness influence rehabilitation strategies. Distal humerus fractures are complex injuries requiring meticulous surgical management to achieve optimal outcomes. This prospective study evaluated 31 patients with closed distal humerus fractures (AO Type 13-C) treated with pre-contoured locking plates, assessing functional recovery using the Mayo Elbow Performance Score (MEPS) over a 6-month follow-up. The mean patient age was 37.5 years, with road traffic accidents being the predominant cause of fracture. MEPS scores improved significantly from 45.97 at 2 weeks to 72.58 at 6 months, with nearly half of the patients achieving good functional results. Common complications included stiffness, screw prominence, nerve compression and infection. Thus, we show that delayed mobilization following bi-columnar plating provides satisfactory outcomes in distal humerus fractures.

## Background:

Distal humerus fractures are complex injuries that account for approximately 2% of all fractures and 33% of all humerus fractures with a bimodal distribution affecting both young males and elderly females [1,2]. Over the past several decades, the management of the distal humerus fractures has undergone substantial evolution. Open reduction and internal fixation has emerged as the standard treatment modality in the majority of displaced intra-articular fractures, with primary goal of achieving anatomical reduction of the articular surface, stable fixation and restoration of elbow function [3]. However, successful management remains technically demanding due to factors such as comminution of the articular surface, metaphyseal bone loss, osteoporotic bone quality and the inherent tendency of the elbow joint to develop stiffness following surgery [4]. Advances in implant design, particularly the introduction of anatomically pre-contoured locking plates provide angular stability and enhanced fixation in osteoporotic bone, allowing for improved construct rigidity and facilitating postoperative rehabilitation protocol [5]. However, the optimal timing of mobilization after surgical fixation of distal humerus fractures remains a topic of debate [6]. While some studies advocate for early mobilization to prevent stiffness and promote functional recovery, others suggest that delayed mobilization may be beneficial in certain cases, such as complex fractures [7] or poor bone quality where concerns regarding fixation stability exist [8, 9-10]. We prospectively assessed the functional outcomes of patients undergoing delayed mobilization at 2 weeks post-surgery, using the Mayo Elbow Performance Score (MEPS), which evaluate pain, range of motion, stability and functional capacity of elbow joint [11]. Therefore, it is of interest to evaluate the functional outcomes of delayed mobilization following bi-columnar plating in patients with distal humerus fractures, AO Type 13-C, using the Mayo Elbow Performance Score (MEPS).

## Material and Methods:

This prospective study was conducted at the Department of Orthopaedics after obtaining approval from the Institutional Ethical Committee (IEC-84/22). The study aimed to evaluate the functional outcomes of patients with closed fracture distal end humerus (AO Type 13-C) managed surgically with pre-contoured anatomical plates. Patients aged 18-65, both male and female, with closed distal humerus fractures (AO Type 13-C) treated with bi-columnar plating within two weeks of injury were included. Exclusion criteria comprised major comorbidities, previous limb surgery, unstable fractures, neurological or vascular conditions, congenital deformities, psychiatric illness, polytrauma and associated limb injuries. Thirty-one patients were included after obtaining written informed consent. Functional assessment was done using the Mayo Elbow Performance Score (MEPS) at regular follow-up intervals (Figure 1 - see PDF). The surgical technique involved patient positioning in a lateral decubitus position, a midline posterior incision over the distal humerus and olecranon osteotomy approach to visualize the articular surface and both columns of the distal humerus. Fracture reduction and temporary stabilization were achieved with K-wires, followed by intercondylar segment reduction and fixation using 4mm cancellous screws. Pre-contoured anatomical plates were applied in an orthogonal configuration to the medial and lateral columns of the distal humerus. Olecranon osteotomy fixation was done using a 6.5mm cancellous partial threaded screw and washer or tension band wiring. All surgeries were performed by same experienced orthopaedic surgeon who followed a standardized protocol to ensure consistency and minimize variability. The patients were managed post-operatively according to a standardized protocol, which included pain management, wound care and rehabilitation. The post-operative protocol included immobilization with a dorsal splint for initial 2 weeks, pain management with medications, wound care and finger and wrist mobilization exercises. At 2 weeks after suture removal, gradual mobilization of the elbow joint was started, beginning with gentle flexion and extension exercises. The elbow mobilization protocol progressed to active-assisted and active range of motion exercises as tolerated, with strengthening exercises started after 4-6 weeks post-op. The rehabilitation protocol was supervised by experienced physiotherapists who worked closely with the patients to ensure that they followed the protocol correctly. The patients were also educated on the importance of adhering to the rehabilitation protocol to achieve optimal outcomes. Functional outcomes were assessed using the Mayo Elbow Performance Score (MEPS) at regular follow-up intervals of 2 weeks, 6 weeks, 3 months and 6 months. The MEPS is a widely used scoring system that evaluates elbow function based on pain, range of motion, stability and daily function. The scores range from 0 to 100, with higher scores indicating better functional outcomes. Data was analysed using SPSS version 24.0. Descriptive statistics (frequencies, percentages, graphs, mean, median, IQR and standard deviation) were used to present results. Statistical significance was tested at a 5% level (p-value). Chi-square tests were used for categorical variables and repeated measures ANOVA was used to compare MEPS at each follow-up. Experienced statisticians, blinded to study outcomes, performed the analysis.

## Results:

The study included 31 patients with closed distal humerus fractures (AO Type 13-C). The mean age was 37.52 ± 13.55 years, with a median of 35 years and a range of 20-65 years. Males comprised 54.8% of the participants, while females made up 45.2%. The mean time since injury was 3.03 ± 1.49 days, with a median of 3 days and a range of 1-7 days. Road traffic accidents (RTA) accounted for 74.2% of injuries, followed by falls from height (FFH) at 25.8% (Table 1 - see PDF). The functional outcomes of the study participants were assessed using the Mayo Elbow Performance Score (MEPS). The mean MEPS score improved significantly over time, from 45.97 ± 8.50 at 2 weeks to 72.58 ± 6.56 at 6 months. The scores at different follow-up intervals were as follows: 45.97 ± 8.50 at 2 weeks, 56.29 ± 7.29 at 6 weeks, 64.84 ± 5.55 at 3 months and 72.58 ± 6.56 at 6 months. A statistically significant association was found between the MEPS score at 2 weeks, 6 weeks, 3 months and 6 months (p = 0.001) (Table 2 - see PDF). The distribution of MEPS categories at different follow-up intervals was also assessed. At 2 weeks, 96.8% of the study participants had poor MEPS and 3.2% had fair MEPS. At 6 weeks, 74.2% of the study participants had poor MEPS and 25.8% had fair MEPS. At 3 months, 38.7% of the study participants had poor MEPS, 45.2% had fair MEPS and 16.1% had good MEPS. At 6 months, 3.2% of the study participants had poor MEPS, 48.4% had fair MEPS and 48.4% had good MEPS (Table 3 - see PDF). The study also assessed the complications that occurred in the study participants. The most common complication was stiffness, which was reported by 19.4% of the study participants. Other complications included screw prominence (9.7%), nerve compression (6.5%) and infection (6.5%). A small proportion (3.2%) of the study participants had implant failure. None of the patients developed myositis (Figure 2 - see PDF). The association between MEPS and mechanism of injury was also assessed. However, no statistically significant association was observed between MEPS at 2 weeks, 6 weeks, 3 months and 6 months and the mechanism of injury (p > 0.05). This suggests that the mechanism of injury did not have a significant impact on the functional outcomes of the study participants (Table 4 - see PDF).

## Discussion:

Distal humerus fractures present unique management challenges due to their intra-articular nature and complex fracture patterns. While treatment options have evolved from casting to internal fixation and arthroplasty, internal fixation remains the preferred approach for achieving optimal outcomes as it allows for precise reduction and stable fixation, which are crucial for restoring joint function [4]. However, fractures with multiple fragments or articular surface damage can complicate the process. In our study, delayed mobilization after surgical management of distal humerus fractures led to satisfactory functional outcomes, with mean MEPS scores improving significantly from 45.97 ± 8.50 at 2 weeks to 72.58 ± 6.56 at 6 months. These results align with Japatti and Janardhan [7] (pre-contoured plates effective), Gupta *et al.* [9] (53.33% excellent, 36.33% good with 2-week stabilization) and Patel and Gadodia and Mistry *et al.* [8, 10] (25% excellent, 50% good but 15% infection/non-union), through our protocol showed lower complications (6.5% infection, 3.2% failure). Delayed mobilization enhanced stability in complex cases, reducing fixation failure risk versus early protocols. The timing of mobilization after distal humerus fracture management is crucial. Initiating mobilization at two weeks, before soft callus formation is complete, strikes a balance between allowing initial healing and preventing complications from prolonged immobilization. This approach prevents fibrosis and stiffness, reduces inflammation, alleviates patient apprehension that can lead to increased pain, spasm and reluctance to participate in physiotherapy and fosters a conductive environment for healing. By doing so, it optimizes patient outcomes, balances stability and movement and minimizes risks, ultimately ensuring effective healing. Our study suggests that delayed mobilization may be a viable option for patients with complex distal humerus fractures, particularly those with comminuted fractures or poor bone quality. Despite the delayed mobilization protocol, the final Mayo Elbow Performance Score (MEPS) at 3 months was comparable to those reported in studies with early mobilization protocols, indicating that delayed mobilization may not compromise functional outcomes. This is supported by a study by Saini *et al.* [12] which found excellent results in 5 patients, good results in 7 patients, fair results in 6 patients and poor results in 2 patients with delayed rehabilitation. Our results at 6 months were consistent with Jadish *et al.* [13] which favored early mobilization, with a similar distribution of MEPS outcomes: 47% excellent, 33% good, 13% fair and 7% poor. Notably, delayed mobilization reduced the risk of fixation failure, as supported by studies by Dayanand and Ezhil [14] and Mahajan *et al.* [15] making it a potentially beneficial approach for patients with complex fractures. The implications of our study are significant, as they suggest that delayed mobilization may be a viable option for patients with complex distal humerus fractures. Study by Kaarlo *et al.* [16] found that ORIF with locking plates for AO/OTA C-type distal humerus fractures in patients over 65 resulted in good outcomes, but with a notable complication rate, including 11 patients requiring revision surgery. This highlights the potential risks of early mobilization in complex fractures. Our study's findings support the consideration of delayed mobilization protocols to minimize complications, particularly in patients with comminuted fractures or poor bone quality. Similar findings were also quoted in other studies [8, 17]. Our study has several limitations, including a small sample size, limited generalizability, potential measurement bias and confounding variables. The single-center design may limit applicability to other settings or populations. Additionally, the limited follow-up duration may not capture long-term outcomes and complications and the lack of a control group receiving early mobilization limits comparison of outcomes. These limitations highlight the need for further research with larger sample sizes, longer follow-up durations and more diverse patient populations to confirm the findings and establish clear guidelines for the timing of mobilization in patients with distal humerus fractures.

## Conclusion:

Delayed mobilization after surgical management of distal humerus fractures yields satisfactory functional outcomes, improving Mayo Elbow Performance Score over time. This approach is beneficial for complex fractures or those with poor bone quality, minimizing complications like fixation failure. While early mobilization prevents stiffness, delayed mobilization can provide comparable functional outcomes, making it a viable option. Further research is needed to establish clear guidelines for mobilization timing in patients with distal humerus fractures.

## Advancement to knowledge:

This study contributes additional clinical evidence regarding the optimal timing of postoperative mobilization following surgical fixation of distal humerus fractures. While most rehabilitation protocols emphasize early mobilization to reduce stiffness, there remains limited prospective data evaluating the outcomes of delayed mobilization after stable bi-columnar plating. The present study demonstrates that initiating elbow mobilization at two weeks post-operatively following fixation with pre-contoured locking plate's results in satisfactory functional outcomes with progressive improvement in Mayo Elbow Performance Score (MEPS) and a relatively low complication rate. These findings suggest that delayed mobilization may provide adequate fracture stability and does not compromise short-term functional recovery in patients with complex intra-articular distal humerus fractures. Therefore, this study adds to the existing body of knowledge by supporting delayed mobilization as a safe and effective rehabilitation strategy in selected patients following bi-columnar plating of distal humerus fractures.

## Statements and Declarations:

## Funding:

The authors declare that no funds, grants, or other support were received during the preparation of this manuscript.

## Ethical approval:

This study was performed in line with the principles of the Declaration of Helsinki. Approval was granted from the Ethics Committee of our Institution (IEC No.84/22 dated August 22, 2022).

## Consent to participate:

Written informed consent has been obtained from the patients for participation and publication.
